# Cirrhotic Cardiomyopathy: Bridging Hepatic and Cardiac Pathophysiology in the Modern Era

**DOI:** 10.3390/jcm14175993

**Published:** 2025-08-25

**Authors:** Dragoș Lupu, Camelia Cornelia Scârneciu, Diana Țînț, Cristina Tudoran

**Affiliations:** 1 Department of Fundamental, Prophylactic, and Clinical Disciplines, Transilvania University of Brasov, 500036 Brașov, Romania; dragos182@yahoo.com (D.L.); scarneciu.camelia@gmail.com (C.C.S.); 2 ICCO Clinics Brasov, 500059 Brașov, Romania; 3 Clinical County Emergency Hospital Brașov, 500157 Brașov, Romania; 4 Department of Medical and Surgical Specialities, Transilvania University of Brașov, 50036 Brașov, Romania; 5 Department VII, Internal Medicine II, Discipline of Cardiology, University of Medicine and Pharmacy “Victor Babes” Timisoara, E. Murgu Square, Nr. 2, 300041 Timisoara, Romania; tudoran.cristina@umft.ro; 6 Centre of Molecular Research in Nephrology and Vascular Disease, University of Medicine and Pharmacy “Victor Babes” Timisoara, E. Murgu Square, Nr. 2, 300041 Timisoara, Romania; 7 County Emergency Hospital “Pius Brinzeu”, L. Rebreanu, Nr. 156, 300723 Timisoara, Romania

**Keywords:** cirrhotic cardiomyopathy, hepatic cirrhosis, physiopathology, galectin 3, nitric oxide

## Abstract

Cirrhotic cardiomyopathy (CCM) is a cardiac dysfunction in patients with cirrhosis, occurring in the absence of structural heart disease. It increases perioperative risk, especially in liver transplantation, and may contribute to hepatorenal syndrome. Despite its clinical significance, CCM remains poorly understood and lacks effective treatments. This review aims to summarize recent findings on the pathogenesis of CCM and highlight potential therapeutic targets. A focused literature review was conducted using PubMed, Scopus, and Clarivate databases, selecting studies from the last five years. Included studies investigated molecular, cellular, and receptor-mediated mechanisms involved in CCM. Results: CCM results from neurohumoral, inflammatory, and electrophysiological disturbances. Key mechanisms involve dysfunction of β-adrenergic and muscarinic receptors, altered ion channels (potassium, L-type calcium), impaired sodium–calcium exchange, and suppression of the P2X7 receptor (P2X7R). Dysregulation of the CD73 (5’-nucleotidase, ecto-5’-nucleotidase)–A2 adenosine axis, along with effects from endocannabinoids, nitric oxide (NO) inhibition by tumor necrosis factor α (TNF-α) and interleukin-6 (IL-6), carbon monoxide (CO), and elevated galectin-3 (Gal-3), further contribute to myocardial dysfunction. Conclusions: CCM is a multifactorial condition linked to systemic and myocardial effects of cirrhosis. A deeper understanding of its mechanisms is essential for developing targeted therapies. Further research is needed to improve patient outcomes.

## 1. Introduction

Cardiovascular changes in patients with end-stage liver disease were first identified approximately 70 years ago. Traditionally, morphological findings included cardiac chamber dilation, cardiomyocyte edema, and myocardial fibrosis, all occurring without ischemic coronary disease, valvular disorders, or arterial hypertension [1]. It was shown that patients with hepatic cirrhosis (HC) exhibit elevated cardiac output, increased peripheral vascular resistance, and subsequently low blood pressure. Despite the heightened cardiac output, their heart response to physiological and pharmacological stimuli is impaired. These cardiac abnormalities, identified three decades ago, were defined as cirrhotic cardiomyopathy (CCM) [2].

Initially, these changes were considered the result of latent alcoholic cardiomyopathy, determined by the cardiotoxic effects associated with toxic nutritional cirrhosis. However, over the past three decades, it has become evident that an abnormal ventricular contractile response to various stimuli can occur, even in patients with non-alcoholic cirrhosis [3].

Based on previous research, CCM is defined by the following:

- Increased cardiac output at rest with altered ventricular contractile response to various stimuli;
- Systolic and diastolic dysfunction;
- Absence of standalone ventricular dysfunction at rest;
- Electrophysiological changes (chronotropic incompetence and prolonged corrected QT interval) [4,5].

The occurrence of CCM in patients with HC is a proven negative prognostic event. Numerous studies conducted over the past 30 years have demonstrated that it is a predisposing factor for the onset of significant complications of cirrhosis, such as cirrhosis decompensation, hepatorenal syndrome, and increased morbidity and mortality post-surgery, post-transplant, and post-infections. Therefore, early diagnosis becomes important [6,7,8].

The pathophysiology of the cardiovascular system in cirrhosis is complex. In both cirrhotic patients and animal models, structural liver abnormalities lead to portal venous hypertension and intestinal congestion, which in turn increases gut permeability. These changes promote bacterial overgrowth, translocation, and the presence of bacteria-derived products in the circulation—such as endotoxins—that stimulate the release of systemic pro-inflammatory cytokines, including TNFα, IL-1β, and IL-6. As a result, individuals with cirrhosis and portal hypertension exhibit a systemic inflammatory profile, contributing to elevated levels of vasodilators such as nitric oxide (NO), carbon monoxide, prostaglandins, bile acids, and glucagon. Consequently, this leads to peripheral vasodilation [9].

The heightened activity of the sympathetic nervous system (SNS) and the elevated serum levels of vasoconstrictive catecholamines in cirrhosis represent a compensatory response to systemic vasodilation rather than its underlying cause. In cirrhotic patients, vasodilatory factors outweigh vasoconstrictive influences, resulting in a characteristic phenotype of systemic vasodilation. Due to this baseline peripheral vasodilation, cardiac dysfunction often remains subclinical at rest, making it difficult to detect in clinically stable individuals. However, when exposed to cardiovascular stressors—such as liver transplantation, transjugular intrahepatic portosystemic shunt insertion, hemorrhage, or administration of vasoactive medications—cardiac dysfunction becomes clinically apparent [10].

Unfortunately, without the existence of a clear diagnostic guide or protocol, the exact prevalence of CCM remains unknown, mainly due to the fact that this condition is generally asymptomatic and the symptoms develop only when the patient is exposed to an external trigger (physical exertion, changes in body position, certain medications, or events like bleeding or surgery).

The reported prevalence of CCM in patients with HC varies depending on the diagnostic criteria. In 2021, in his meta-analysis, Shahvaran et al. found a prevalence of 61% using the 2005 criteria and 41% with tissue Doppler-based criteria [11]. Their findings indicate that reliance on conventional two-dimensional echocardiography in conjunction with the 2005 Montreal criteria may result in the overdiagnosis of diastolic dysfunction (DD) and, consequently, CCM. Moreover, tissue Doppler imaging (TDI) and standard two-dimensional echocardiography demonstrate poor concordance in diagnostic accuracy, further highlighting the limitations of these conventional modalities in reliably identifying CCM [11]. Similarly, Razpotnik et al. (2021) reported comparable prevalence rates using the 2005 Montreal criteria (67%) and the 2019 Cirrhotic Cardiomyopathy Consortium (CCC) criteria (55%, *p* = 0.09) [12]. In his study from 2021, Cesari et al. retrospectively analyzed a dataset comprising 162 cirrhotic patients who were followed for a minimum of six years [13]. All patients underwent standard Doppler echocardiographic evaluation and were compared with a control group of 46 healthy subjects. Left ventricular (LV) systolic (assessed by global longitudinal strain (GLS), ejection fraction, and fractional and midwall fractional shortening) and diastolic function (assessed by early and late diastolic filling velocities, their ratio, the early wave deceleration time, and the presence of LV hypertrophy), according to the current guidelines recommendations. No patient exhibited an LVEF ≤ 50%. A global longitudinal strain (GLS) value of <18% or >22% was observed in 25% of patients, while severe diastolic dysfunction was identified in 10%. The authors determined an incidence of 20% for CCM primarily due to abnormal resting GLS. However, to improve the prognostic relevance of their results, the authors suggest including stress assessment of the cardiac contractile reserve [13]. In 2023, Karagiannakis et al. demonstrated that the prevalence of left ventricular diastolic dysfunction was significantly lower with the 2019 criteria (11%) compared with the 2005 and 2009 criteria (80% and 40%, respectively) [14].

Based on the studies mentioned above, the available data show significant variability across different patient populations, making it difficult to establish the precise prevalence of CCM.

The aim of this review is to provide a comprehensive and up-to-date overview of CCM, with a particular focus on its underlying pathogenic mechanisms, as reported in the most recent scientific literature. Our article is distinctive, as it analyzes all the mechanisms involved in the pathophysiology of CCM. It is structured in two parts: the first part addresses the mechanisms that are already well established, while the second part focuses on research conducted over the past five years. This includes, in particular, studies related to the P2X7 receptor, the protective role of the CD73 (5’-nucleotidase, ecto-5’-nucleotidase)/A2AR3 axis, the inhibitory effect of galectin-3 on myocardial contractility, and the role of beta-adrenergic receptors, calcium channels, and muscarinic receptors, as well as the involvement of cannabinoid receptors and their agonists in mitigating cardiac inflammation and fibrosis.

## 2. Established Pathophysiological Mechanisms of Cirrhotic Cardiomyopathy

The mechanisms of the development of CCM are only partially understood, despite numerous studies conducted on both animals and humans. These mechanisms are diverse and complex, involving neurological, humoral, and vascular disturbances.

In cirrhosis, fibrosis and the destruction of the liver’s normal architecture increase vascular resistance in the liver, a phenomenon some researchers refer to as retrograde portal hypertension. On the other hand, substances such as NO, carbon monoxide (CO), glucagon, and other vasodilators dilate the mesenteric vascular bed, forming what is known as hyperdynamic splanchnic circulation, or forward portal circulation [15].

Together, these processes contribute to mesenteric vascular congestion, which facilitates intestinal bacterial translocation and, subsequently, endotoxemia. Endotoxemia stimulates the secretion of pro-inflammatory cytokines, such as TNFα and IL-1β, both of which inhibit myocardial contractility [16].

Hyperdynamic circulation likely originates from peripheral and splanchnic vasodilation, leading to a reduced effective arterial circulating volume. This reduction in turn decreases renal perfusion, activating the renin–angiotensin–aldosterone system (RAAS), the sympathetic nervous system (SNS), and the release of antidiuretic hormone. These stimuli further result in renal arterial vasoconstriction and sodium and water retention [17].

As liver function progressively deteriorates, systemic vasodilatation becomes more pronounced. This intensifies the hyperdynamic circulation and aggravates renal vasoconstriction, thereby amplifying the aforementioned effects [18].

For a better understanding of the pathophysiological mechanisms, they can be divided into two categories: vascular changes and cardiac changes.

### 2.1. Vascular Changes

Arterial vasodilation in cirrhosis is a multifaceted process involving interactions between various vasodilator and vasoconstrictor molecules and the vascular endothelium. In cases of hepatic cirrhosis complicated by portal hypertension, the formation of portosystemic collateral vessels occurs, which bypass the liver and allow certain substances to pass directly into the systemic circulation without being detoxified by the liver.

In recent years, NO has been recognized as the primary vasodilator in both the splenic and systemic circulations. It is produced in increased quantities in patients with cirrhosis, and its distribution differs in the splenic circulation [19,20].

Intrahepatic circulation is notably disrupted due to structural damage and biochemical changes in this region, which increases the secretion of vasoconstrictors such as angiotensin 1, endothelin 1, and cysteinyl-leukotrienes, accompanied by a decrease in intrahepatic NO concentration. The result of these changes is increased intrahepatic vascular resistance and portal hypertension [21].

On the other hand, it is well-documented that in other parts of the splenic circulation, NO production is elevated, possibly due to the transfer of endotoxins from the intestinal membrane directly into the systemic circulation. These endotoxins promote NO production [22].

Another molecule that induces NO production is TNFα, as proven by numerous animal studies. Inhibition of TNFα production using specific antibodies has led to normalization of NO production and, consequently, the normalization of hyperdynamic circulation [23,24].

The enzyme responsible for NO synthesis is nitric oxide synthase (NOS). Three isoforms of this enzyme have been described: endothelial (NOS 3), inducible (NOS 2), and neuronal (NOS 1). It is still unclear which of these isoforms is dominant, but several clinical studies that used NOS inhibitors have shown that reducing NO production resulted in normalization of peripheral vascular vasodilation [25].

Ferguson et al. demonstrated that selective inhibition of NOS 2 led to peripheral vasoconstriction in patients with cirrhosis, an effect not observed in individuals without the condition. Additional studies indicate that NOS 3 is the primary source of NO production in the splenic circulation [26,27].

Furthermore, endocannabinoids have also been identified as key contributors to peripheral vasodilation [27]. These are lipid molecules that act on two G-protein-coupled receptors, known as CB1 and CB2. The activation of CB1 receptors by endogenous endocannabinoids caused pronounced vasodilation in cirrhotic rats included in the study, as was demonstrated by Batkai et al. [28].

### 2.2. Cardiac Changes

**Figure 1:**
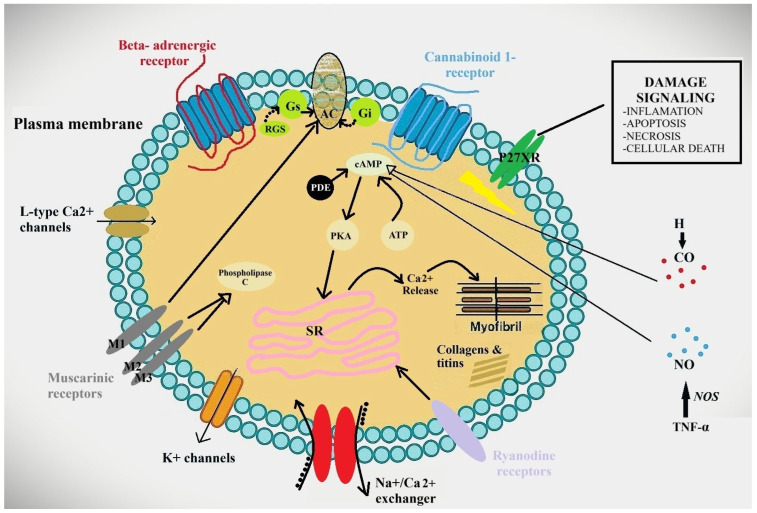
Changes in the function of K^+^, L-type Ca^2+^, Na^+^/Ca^2+^ channels, and beta-adrenergic and cannabinoid receptors occurring in CCM. AC—adenylyl cyclase; ATP—adenosinetriphosphate; Ca^2+^—calcium; CO—carbon monoxide; cAMP—cyclic adenosine monophosphate; Gs—stimulatory G protein; Gi—inhibitory G protein; H—hydrogen; K^+^—potassium; PDE—phosphodiesterase; PKA—protein kinase A; NO—nitric oxide; NOS—nitric oxide synthase; SR—sarcoplasmic reticulum; TNF—tumor necrosis factor.

The changes in the heart are complex and intricate (Figure 1). Several potential molecular causes have been identified over time to explain the reduction in myocardial function. These include the following:

1. Changes in cardiomyocyte membrane structure;
2. Changes at the level of myocardial receptors;
3. Alterations in ion channel function;
4. Molecular mediators.

#### 2.2.1. Changes in Cardiomyocyte Membrane Structure:

In a study conducted on rats with bile duct ligation, Ma et al. demonstrated an increase in cholesterol levels in the cardiomyocyte membrane, which leads to a decrease in membrane fluidity. As a result, there was a reduction in cyclic adenosine monophosphate (cAMP) production in response to sympathetic nervous stimulation. This finding was reversible upon restoration of the membrane’s normal fluidity [29].

In a separate study, it was found that bile acid reduces cardiomyocyte membrane fluidity, leading to the same results of reduced cAMP levels [30].

#### 2.2.2. Changes at the Level of Myocardial Receptors

##### Alteration of Beta-Adrenergic Receptor Density and Function

In patients with CCM, a decrease in the heart’s chronotropic and inotropic responses to adrenergic stimulation has been described. This condition can be explained by a reduction in the density of beta-adrenergic receptors and a decrease in their function. These alterations have been observed in several experimental studies conducted on animals [31,32].

##### Changes in Muscarinic Receptor Function

Acetylcholine receptors are divided into two subtypes: muscarinic and nicotinic. Muscarinic receptors are further divided into five subtypes. Receptors M1, M3, and M5 stimulate phospholipase C, while M2 and M4 receptors inhibit adenylate cyclase.

In terms of location, M2 and M3 receptors are found in cardiac tissue, while M1, M2, and M3 receptors are located in the vascular endothelium. In the heart, most muscarinic receptors are found in the atria and are more prevalent in the endocardium compared with the epicardium [33]. These receptors are also located in cardiomyocyte T-tubules, coronary arteries, and capillary endothelium, as well as in the sinoatrial and atrioventricular nodes [34]. Muscarinic receptors exert antagonistic effects on adrenergic receptors, as described in multiple studies.

Jaue et al. showed that there is a decreased M2-mediated muscarinic response and a decreased production of cAMP in rats with hepatic cirrhosis [35].

#### 2.2.3. Alterations in Ion Channels’ Function

##### Changes in Potassium Channels

Potassium channels are activated by decreased cytoplasmic levels of adenosine triphosphate (ATP) and function as voltage-independent inhibitors in the depolarization of the cardiomyocyte [36].

Their activation is essential for both early and late repolarization processes. Activators of these channels (such as adenosine) induce hyperpolarization and thus relaxation, while inhibitors (such as norepinephrine, angiotensin II, and endothelin-1) induce depolarization and contraction. In comparison to healthy animals, the findings of a study on cirrhotic animals revealed a lower potassium channel current density, which caused ventricular cardiomyocytes to experience an action potential length that was longer [37]. The extended QT interval frequently seen in CCM patients may be explained by these findings. Moreover, substantial action potential prolongation might maintain the cardiomyocyte in a condition of prolonged contraction, which would impact ventricular relaxation [38].

Additionally, Nichols et al. identified a key potassium channel responsible for regulating the resting membrane potential in cardiac cells, which also plays a direct role in the final phase of repolarization. Subsequent research revealed that this channel is essential for maintaining inotropic function, and its dysfunction may result in diminished inotropic capacity [39].

##### Changes in L-Type Calcium Channels

In cardiac myocytes, membrane depolarization opens L-type calcium channels, triggering calcium release from the sarcoplasmic reticulum through ryanodine receptors. Phosphorylation of these receptors and a decrease in calcium from the sarcoplasmic reticulum can reduce the total amount of calcium available for depolarization [40].

Ward et al. reported that during myocardial relaxation, calcium is reabsorbed into the sarcoplasmic reticulum and expelled into the extracellular space via ATP-dependent calcium pumps and sodium/calcium exchangers. Their study found a decrease in both the initial calcium influx and its subsequent release from the sarcoplasmic reticulum [41].

Another study conducted on cirrhotic patients has found a decrease in the density of cardiac L-type calcium channels and a reduction in sarcoplasmic calcium with similar effects [42].

##### Alteration of Normal Functioning of the Sodium-Calcium Exchanger

The sodium/calcium exchanger is essential for regulating the balance between calcium entering and exiting the cell. Found in nearly all cell membranes, it operates by exchanging three sodium ions for one calcium ion. This exchanger is regarded as the primary mechanism for controlling intracellular calcium levels, while a secondary mechanism involves calcium transport through the ATP-dependent sarcolemmal pump. Since excess calcium influx leads to cardiomyocyte apoptosis [43], malfunctions in this exchanger could play an important role in the development of CCM. However, this hypothesis requires validation through further studies.

#### 2.2.4. Molecular Mediators

**Figure 2:**
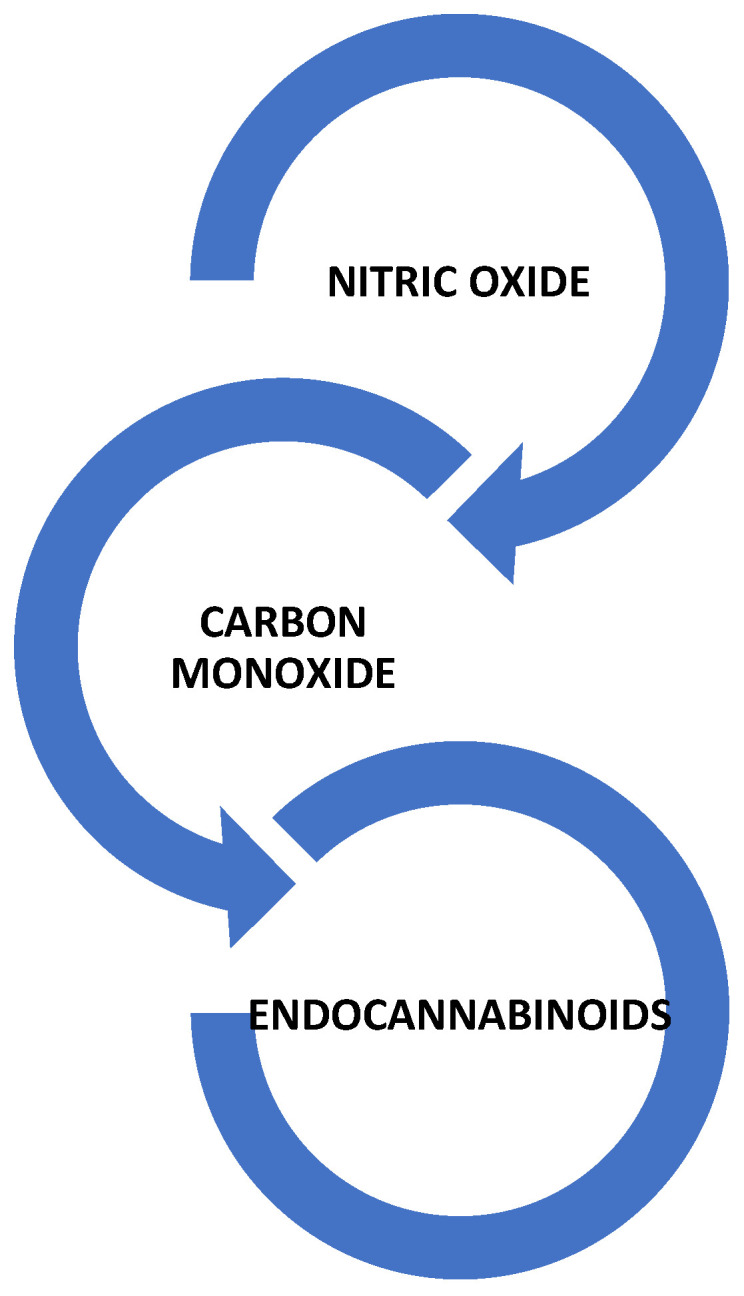
The main molecular mediators involved in the pathophysiology of CCM.

There are several molecular mediators involved in the development of CCM, as shone in Figure 2.

##### Carbon Monoxide

CO is an endogenously produced, short-lived gas that induces vasodilatation in the splenic arteries. In hepatic cirrhosis, CO production can be stimulated via activation of the sympathetic nervous system, elevated norepinephrine levels, or increased cytokine release due to endotoxemia.

CO reduces ventricular contractility by increasing cyclic guanosine monophosphate (cGMP) levels and decreasing calcium influx. In a study on rats with bile duct ligation, Liu et al. demonstrated that the activation of the heme oxygenase–carbon monoxide (HO–CO) pathway occurs in the hearts of cirrhotic rats, as evidenced by upregulated HO-1 expression. Inhibition of CO signaling was associated with reduced production of the second messenger cGMP and improved cardiac contractility. These observations suggest that CO exerts negative inotropic effects through cGMP-mediated signaling. Collectively, the data support a role for increased CO production—resulting from HO pathway activation—in the pathogenesis of CCM [44].

##### Endocannabinoids

Endocannabinoids induce splenic arterial vasodilation and exert negative inotropic effects at the cardiac level. These are lipid compounds that bind to specific receptors (CB1 receptors—found in the brain, heart, hepatic sinusoidal cells, smooth muscle cells, and peripheral nerves—and CB2 receptors, which are located in immune cells) [45].

In a study involving cirrhotic animal models, Gaskari et al. demonstrated that the ventricular response to beta-adrenergic stimulation is suppressed due to elevated endocannabinoid levels, which act through CB1 receptor activation [46]. Similarly, another study using animals with carbon tetrachloride-induced hepatic cirrhosis found that increased endocannabinoid activity via CB1 receptor stimulation adversely affected cardiac contractility [47]. This negative inotropic effect of CB1 receptor activation may be attributed to the inhibition of L-type calcium channels, leading to a subsequent reduction in cAMP levels.

##### Nitric Oxide

Preliminary findings have shown that NO-induced hyperdynamic circulation in patients with HC, as well as splenic arterial vasodilation, masks altered cardiac function, thereby reducing afterload [48,49].

NO is synthesized in coronary endothelial cells and cardiomyocytes through the activity of NOS, which converts L-arginine to L-citrulline. Two sub-enzymes are present in cardiomyocytes: NOS3, located in the cytoplasmic caveolae, and NOS1, found in the sarcoplasmic reticulum [50].

The third isoform, NOS2, can only be expressed following stimulation by inflammatory mediators.

NO produced by NOS3 and NOS1 exerts several cardioprotective effects, including improved coronary perfusion and anti-apoptotic effects. Its release is pulsatile and influenced by variations in ventricular filling pressure, which subsequently modulate the activity of ion channels involved in excitation-contraction coupling [51].

Preliminary observations have shown that NO-mediated hyperdynamic circulation and splenic arterial vasodilation in patients with HC reduce afterload, thereby masking underlying cardiac dysfunction [52]. Experimental studies in cirrhotic animal models have further elucidated the role of NO and NOS in the pathophysiology of CCM.

Van Obbergh et al. were the first to establish a link between NOS and CCM, demonstrating that inhibition of NOS using NG-Monomethyl-L-arginine (L-NMMA) significantly improved ventricular contractility in cirrhotic rats [53]. Supporting this, Liu et al. reported elevated levels of NOS2 in the hearts and NOS3 in the aortas of cirrhotic rats. They found that increased NO levels exert a cardiodepressant effect, further implicating NO in CCM pathogenesis [54].

The mechanism of NO’s action involves activation of soluble guanylate cyclase, resulting in a dramatic increase of up to 400-fold in cGMP levels. Elevated cGMP can inhibit L-type calcium channels and reduce the responsiveness of conduction system cells to adrenergic stimulation, ultimately contributing to bradycardia and impaired chronotropic function [55].

Inflammatory cytokines also appear to mediate cardiac dysfunction via NO-dependent pathways. In one study, both TNF-α and IL-1β exerted negative inotropic effects on the papillary muscles of cirrhotic rats, an effect that was mitigated when NO production was inhibited, highlighting NO’s central role in CCM [56].

Additionally, NO contributes to oxidative stress in the myocardium through the formation of peroxynitrite, a reactive nitrogen species generated by the reaction of NO with superoxide anion. Peroxynitrite can nitrosylate cardiac contractile proteins, thereby impairing their function [54]. This process was further explored by Mani et al., who found that protein nitrosylation in cardiac tissue was associated with diminished chronotropic response in cirrhotic animals [57].

Together, these findings underscore the multifaceted role of NO in the pathogenesis of CCM, involving vascular alterations, impaired calcium signaling, inflammatory cytokines, and oxidative stress.

##### Role of Apoptosis in Alteration of Cardiac Function in Patients with Cirrhotic Heart Disease

Apoptosis is a key cellular mechanism involved in myocardial remodeling associated with heart failure. Among the major signaling pathways regulating apoptosis, mitogen-activated protein kinases (MAPKs) play a crucial role in mediating cellular responses to a wide range of physiological and pathological stimuli. Among the MAPK subtypes, p38 MAPK is notably implicated in cellular proliferation, differentiation, and apoptosis [58,59]. It is well established that chronic hepatic conditions activate p38 MAPK in myofibroblasts, although the exact mechanisms underlying this activation are not yet fully understood [60]. Genetic studies have demonstrated that the p38 MAPK isoform contributes to myocardial cell death following ischemic injury [61]. Furthermore, the use of a selective p38 MAPK inhibitor has shown protective effects at the myocardial level post-ischemia, reinforcing the pro-apoptotic role of this isoform [62]. Consequently, the activation of p38 MAPK in HC may promote myocardial apoptosis, playing a pivotal role in the progression of cardiac dysfunction. This apoptotic pathway contributes to the loss of myocardial cells and exacerbates myocardial remodeling, highlighting the multifactorial nature of heart failure in cirrhotic patients [63].

## 3. Emerging and Novel Mechanisms in Cirrhotic Cardiomyopathy

### Selection Process of State-of-the-Art Original Articles

**Figure 3:**
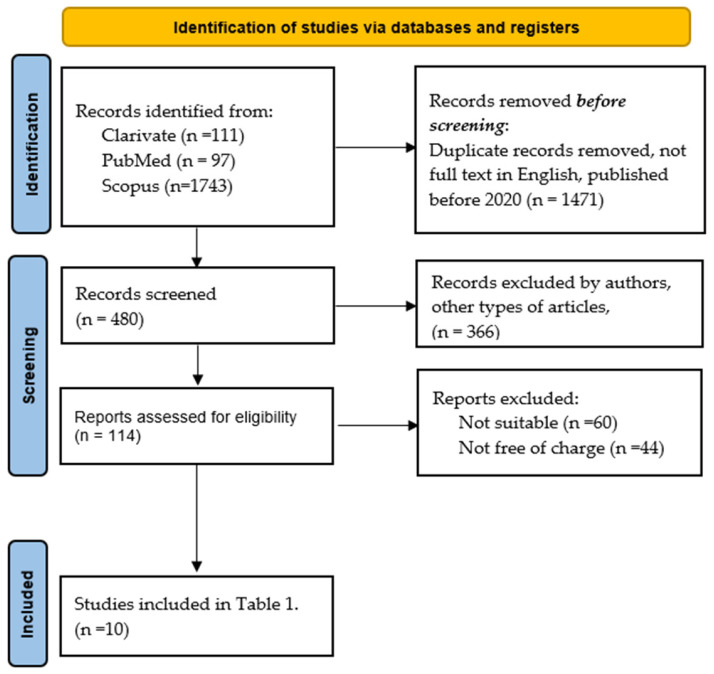
PRISMA flow diagram.

To identify the most relevant articles for our review, we searched PubMed, Scopus, and Clarivate using “Cirrhotic cardiomyopathy mechanisms” as a keyword. We obtained a total of 1951 manuscripts. We used Zotero to remove duplicate records. Predefined database filters were applied to exclude all article types except for original research articles, cohort studies, and case and control studies. As our goal was to analyze in this section only the latest research on mechanisms involved in the development of CCM, we selected original articles published between 2020 and 2025. Other selection criteria were full-text articles available in English, free of charge. Following this selection, the remaining 37 researches were assessed for eligibility. An additional 114 manuscripts were excluded either because they were not freely accessible or were deemed irrelevant for our review. As a result, 10 articles were included in the final analysis (see Figure 3).

The data on the physiopathological mechanisms of CCM from all nine relevant articles are presented in detail in Table 1.

A study conducted by Sun et al. in 2025 aimed to provide new insights into the complex interplay between cirrhosis and cardiac dysfunction, offering a possible explanation for the development of cardiac fibrosis in patients with CCM [64]. By analyzing cytokine profiles in the peripheral blood, the authors observed that elevated circulating TGF-β1 may play a pivotal role in modulating cardiac remodeling through epigenetic regulation. Specifically, the results suggested that increased TGF-β1 levels can inhibit Notch1 (neurogenic locus notch homolog protein 1) signaling via activation of the DNMT3A (DNA methyltransferase 3 alpha)/methyl-CpG binding protein 2 (MeCP2) pathway, leading to enhanced Notch1 promoter methylation and subsequent cardiac fibrosis [64].

In a study conducted by Ma et al. on human subjects, it was demonstrated that the reduced cardiac contractility observed in cirrhosis has been linked to decreased membrane fluidity and impaired beta-adrenergic receptor signaling. These findings suggest that in cirrhotic cardiomyopathy, increased membrane rigidity disrupts the coupling between beta-adrenoceptors and G-proteins [73]. Also, in a study conducted on rats, cardiac contractile dysfunction in cirrhosis has been associated with altered β-adrenergic receptor signaling and dysregulated expression of guanine nucleotide-binding proteins. These abnormalities may contribute significantly to the pathogenesis of CCM [73].

Ma et al. concluded in their research from 2021 that anti-β1-adrenergic receptor (anti-β1-AR) antibody levels were significantly elevated in the CCM group compared with the non-CCM group. These findings suggest that anti-β1-AR may serve as a valuable predictive biomarker for the presence of CCM and could potentially have therapeutic implications [73].

Regarding the role of calcium channels, Bayat et al. demonstrated in their 2022 study that animals with CCM experienced significant up-regulation of ventricular β_1_-adrenergic receptors and L-voltage-dependent calcium channels [68].

Research conducted by Yu et al. in 2021 on cirrhotic rats, which focused on IL-6 levels, beta adrenergic receptors, and muscarinic receptors, concluded that in rats with cirrhosis, the levels of the pro-inflammatory cytokine IL-6 were elevated, whereas the expression of β1-AR protein and muscarinic M2 receptors was reduced. These findings suggest that aberrant inflammatory responses, altered autonomic regulation, and other mechanisms may contribute to myocardial injury and accelerate ventricular remodeling in cirrhosis [70]. Also, Gregolin et al. found in their 2021 research that CCM is characterized by reduced cardiac contractility accompanied by altered phospholamban phosphorylation and elevated cardiac pro-inflammatory IL-6 levels [69].

Our review further underscores how the CD73 (ecto-5’-nucleotidase) pathway intersects with core inflammatory and fibrotic mechanisms implicated in the pathogenesis of CCM. Danger-associated molecular patterns (DAMPs) such as ATP are released during cellular injury and necrosis, where they act extracellularly as potent immune-stimulatory signals that activate purinergic receptors and perpetuate inflammatory cascades [74]. In this context, Zhao et al. demonstrated in a CCM mouse model that CD73(5’-NUCLEOTIDASE, ECTO-5’-NUCLEOTIDASE)-mediated hydrolysis of extracellular ATP to adenosine plays a pivotal role in suppressing excessive inflammation and reducing myocardial apoptosis, thereby highlighting the therapeutic potential of CD73(5’-NUCLEOTIDASE, ECTO-5’-NUCLEOTIDASE) in modulating inflammation-associated cardiac injury [66].

These findings align with a broader body of evidence showing that CD73(5’-NUCLEOTIDASE, ECTO-5’-NUCLEOTIDASE)-expressing immune cells dynamically shape the inflammatory milieu by regulating the balance between pro-inflammatory ATP signaling and anti-inflammatory adenosine signaling [75]. For example, in myocardial infarction models, CD73(5’-nucleotidase, ecto-5’-nucleotidase) expression on T cells has been shown to attenuate inflammation by activating A2A adenosine receptors (A2AR), which inhibit the production of pro-inflammatory cytokines and limit tissue damage [76].

Another important role in the pathogenesis of CCM is represented by the increased levels of galectin 3 (Gal-3). Gal-3 serves as a biomarker for inflammation and fibrosis in cardiac tissues and is primarily expressed by activated macrophages. Gal-3 is significantly increased in patients with cirrhosis and fibrotic/cirrhotic animal models [77]. In a 2022 study by Yoon et al., the researchers investigated the role of Gal-3 in cardiomyopathy-related factors and cardiac contractility using a rat model of cirrhotic cardiomyopathy. The study found that elevated levels of Gal-3 in the cirrhotic heart significantly impaired cardiac contractility, primarily through the action of TNFα. Notably, inhibition of Gal-3 led to a reduction in cardiac levels of TNFα and brain natriuretic peptide (BNP), while simultaneously restoring blood pressure and improving myocardial contractility. These findings suggest that Gal-3 plays a pathogenic role in the development and progression of CCM [67].

Honar et al. conducted a study on cirrhotic rats to investigate the role of myosin heavy chain isoform shifts and their relationship to calcium transients in the contractile kinetics of cirrhotic hearts [71]. Their findings revealed that cardiomyocytes and ventricular trabeculae from the cirrhotic rat model exhibited hallmark features of heart failure, including prolonged systolic and diastolic phases, a blunted force–frequency relationship, and reduced force-generating capacity. The study identified dysfunctional myosin isoform switching and altered calcium transients as key mechanisms underlying the heart failure phenotype associated with cirrhosis [71].

The P2X7 receptor (P2X7R) is a type of purinergic receptor involved in inflammation. It has been implicated in the development of various autoinflammatory, autoimmune, chronic inflammatory, and metabolic diseases. Research conducted by Shao et al. in 2024 highlighted the importance of this receptor in the development of CCM. An animal model of cirrhotic cardiomyopathy was developed by performing bile duct ligation in mice. To inhibit (P2X7R) expression, Brilliant Blue G was administered intraperitoneally. Cardiac function was assessed using echocardiography, while inflammation and apoptosis in liver and cardiac tissues were evaluated through histopathological analysis and enzyme-linked immunosorbent assays (ELISA). A significant increase in myocardial inflammation and apoptosis was observed, leading to impaired cardiac function. Notably, P2X7R expression was markedly upregulated in both cardiac and hepatic tissues. Inhibition of P2X7R expression effectively reduced myocardial inflammation and apoptosis, resulting in improved cardiac function. These findings indicate that targeting the P2X7-NLRP3 signaling axis may present a promising therapeutic strategy for the treatment of CCM [65].

Matyas et al. aimed to characterize the detailed hemodynamic profile of mice with bile duct ligation-induced liver fibrosis by assessing echocardiographic parameters, intracardiac pressure–volume relationships, and myocardial structural changes [72]. To investigate the influence of liver inflammation and fibrosis on cardiac function, a selective cannabinoid-2 receptor (CB2-R) agonist—known to attenuate inflammatory and fibrotic responses—was administered. The results showed that activation of CB2-R significantly ameliorated hepatic inflammation, microcirculatory impairment, and fibrosis. Furthermore, CB2-R stimulation reduced serum TNF-α levels and improved cardiac dysfunction, myocardial inflammation, and oxidative stress. These findings highlight the critical role of inflammatory mediators in the pathogenesis of hepatic cardiomyopathy [72].

## 4. Discussion

CCM remains a multifaceted and incompletely understood complication of advanced liver disease, characterized by subtle yet clinically significant alterations in cardiac structure and function. This review is intended to synthesize the current body of evidence regarding the diverse molecular, cellular, and hemodynamic mechanisms responsible for the development of CCM, with particular emphasis on both well-established pathways and emerging insights from recent research. By critically examining these mechanisms, we aimed to clarify how cirrhosis exerts its detrimental cardiac effects, identify inconsistencies in the literature, and highlight areas that warrant further investigation to allow a more accurate diagnosis and targeted therapeutic strategies.

With regard to the role of beta-adrenergic receptors, the mechanisms contributing to the development of CCM are both complex and multifactorial. Consistent with our research, recent studies by Ryu et al. [78] and Liu et al. [79] have demonstrated similar findings, underscoring the intricate interplay between altered beta-adrenergic signaling and cardiac dysfunction in cirrhosis. Specifically, Ryu et al. reported that chronic sympathetic overactivity in cirrhotic patients leads to beta-adrenergic receptor desensitization and downregulation, thereby impairing myocardial contractility and contributing to the blunted cardiac response to stress [78].

Similarly, Liu et al. highlighted the role of altered beta-adrenergic receptor density and downstream signaling pathways, which exacerbate myocardial dysfunction and increase susceptibility to hemodynamic instability in advanced liver disease [79]. Together, these observations emphasize the pivotal role of beta-adrenergic dysregulation in the pathophysiology of CCM and point to the need for therapeutic strategies that address this maladaptive response. Furthermore, with respect to muscarinic receptors, this review also highlights recent research that has advanced our understanding of their role in the pathogenesis of CCM. Additionally, this our research draws attention to recent advances in the understanding of muscarinic receptors and their involvement in the pathophysiology of CCM. Recent studies have demonstrated that dysregulation of muscarinic receptor signaling may contribute to impaired autonomic balance and exacerbate myocardial dysfunction in cirrhosis, highlighting the need for continued research into their potential as diagnostic or therapeutic targets [79].

The involvement of voltage-gated ion channels in the pathogenesis of CCM is described in both of the aforementioned reviews, as well as in the study by Desai et al. [80]. These channels play a critical role in regulating myocardial excitability and contractility, and their dysfunction in cirrhosis has been associated with altered calcium handling, impaired action potential propagation, and an increased susceptibility to arrhythmias. By disrupting normal ion fluxes, cirrhosis-induced changes in channel expression and function may contribute to the development of both systolic and diastolic dysfunction, further aggravating cardiac performance in affected patients.

Notably, the last five years have witnessed a marked surge in preclinical and clinical studies that have expanded our understanding of the multifaceted pathways underlying myocardial dysfunction in cirrhosis.

Emerging evidence has refined classical concepts of autonomic dysregulation and β-adrenergic receptor desensitization by integrating novel insights into inflammatory mediators, fibrotic remodeling, ion channel dysregulation, and the role of key molecular pathways such as the P2X7 receptor, CD73(5’-nucleotidase, ecto-5’-nucleotidase)–A2AR axis, galectin-3 signaling, and the endocannabinoid system. Collectively, these discoveries highlight the intricate interplay between chronic systemic inflammation, maladaptive neurohumoral responses, and progressive myocardial structural alterations.

The activation of the purinergic–adenosinergic axis may interface with other key pathways involved in cirrhosis-induced myocardial injury, including TGF-β1 signaling, Notch1 suppression through epigenetic regulation, and the activation of P2X7R-mediated inflammatory pathways. Together, these interconnected pathways create a self-amplifying loop of sterile inflammation, fibroblast activation, and maladaptive cardiac remodeling. The ability of CD73(5’-nucleotidase, ecto-5’-nucleotidase) to shift this balance by enhancing local adenosine generation suggests a promising target for interrupting this cycle at multiple levels.

Parallel to these receptor-level changes, inflammation-driven molecular alterations have also emerged as critical contributors. TGF-β1, a well-established profibrotic cytokine, was shown by Sun et al. [64] to regulate cardiac fibrosis epigenetically in cirrhosis. Their work revealed that TGF-β1 suppresses Notch1 signaling through activation of the DNMT3A/MeCP2 epigenetic axis, resulting in Notch1 promoter methylation and pathological cardiac remodeling.

Adding further complexity to CCM pathogenesis, recent studies implicate purinergic signaling and immunometabolic pathways as critical modulators of myocardial injury and repair. CD73, an ecto-5’-nucleotidase that catalyzes the conversion of ATP to adenosine, plays a cardioprotective role by regulating inflammation. Activation of CD73(5’-nucleotidase, ecto-5’-nucleotidase) in a murine CCM model suppressed myocardial apoptosis and reduced pro-inflammatory cytokine production. These effects are mediated via A2A adenosine receptor signaling, which promotes an anti-inflammatory milieu and facilitates tissue repair. The CD73(5’-NUCLEOTIDASE, ECTO-5’-NUCLEOTIDASE)–adenosine axis likely intersects with other key pathways—including TGF-β1–Notch1 epigenetic suppression and P2X7 receptor (P2X7R)-mediated inflammasome activation—to create a self-amplifying loop of fibrosis, inflammation, and cardiac dysfunction.

Galectin-3 (Gal-3) has emerged as a central mediator of myocardial inflammation and fibrosis in cirrhotic cardiomyopathy (CCM), underscoring its dual role as both a pathogenic effector and a potential biomarker. Pharmacologic inhibition of Gal-3 resulted in reduced myocardial levels of TNF-α and brain natriuretic peptide (BNP), along with a marked improvement in systolic function and systemic blood pressure. These findings implicate Gal-3 as a key upstream regulator of the inflammatory-fibrotic axis in CCM, with direct effects on ventricular remodeling. Given its established role in cardiac fibrosis and heart failure in non-cirrhotic populations, Gal-3 may represent a novel therapeutic target in patients with cirrhosis who are at risk of cardiovascular complications. Its detectability in serum further supports its utility as a non-invasive biomarker for early CCM detection and progression monitoring, potentially guiding risk stratification in transplant candidates.

In parallel, myofibrillar remodeling has been identified as a structural correlate of myocardial dysfunction in CCM. Significant shifts in myosin heavy chain isoform expression, alongside abnormalities in calcium transients, contribute to a protracted systolic and diastolic phase, reduced contractile force, and a blunted force–frequency relationship. These alterations mirror the maladaptive remodeling seen in heart failure with preserved ejection fraction (HFpEF), reinforcing the notion that CCM shares pathophysiological features with classic cardiomyopathies. The presence of these cellular-level abnormalities underscores the chronicity and irreversible nature of myocardial involvement in advanced cirrhosis and highlights the urgent need for early detection and therapeutic intervention before irreversible remodeling occurs. Collectively, these findings emphasize that beyond hemodynamic dysregulation, inflammatory, metabolic, and structural changes converge to create a heart failure phenotype in CCM, with Gal-3 and myofibrillar remodeling representing key mechanistic and translational targets for future research.

Cannabinoid receptor signaling has also been shown to influence cardiac function in cirrhosis. Gaskari et al. [46] demonstrated that activation of CB1 receptors in cirrhotic rats leads to suppressed β-adrenergic responsiveness and reduced inotropy. In contrast, CB2 receptor activation, as shown by Matyas et al. [72], ameliorated hepatic inflammation, oxidative stress, and myocardial dysfunction, suggesting differential therapeutic potentials of cannabinoid receptor subtypes.

Taken together, these findings underscore the multifactorial nature of CCM, where structural, neurohumoral, inflammatory, and epigenetic mechanisms converge to promote myocardial dysfunction. Although animal models have been instrumental in elucidating these pathways, limitations persist. Standard models such as bile duct ligation (BDL) or carbon tetrachloride (CCl_4_) exposure do not capture the complexity of human cirrhosis etiologies (e.g., NAFLD, viral hepatitis, alcohol) or associated comorbidities. This etiological mismatch hinders translation of preclinical insights into clinical practice.

Future studies should prioritize the development of comorbidity-inclusive and etiology-specific models, alongside clinical stratification of CCM patients by underlying liver disease type. Integrating mechanistic insights with biomarker discovery—such as anti-β1-AR, Gal-3, CD73(5’-NUCLEOTIDASE, ECTO-5’-NUCLEOTIDASE), and TGF-β1/Notch1 epigenetic profiles—may enable earlier diagnosis and targeted intervention. The expanding recognition of immune-metabolic pathways and receptor-based signaling cascades offers fertile ground for precision medicine approaches in CCM management.

Animal models have been instrumental in elucidating the pathophysiology of cirrhotic cardiomyopathy (CCM). Commonly used models include bile duct ligation (BDL) and carbon tetrachloride (CCl_4_) administration in rodents, which reliably induce cirrhosis and its hemodynamic sequelae [81]. However, several limitations restrict the translatability of these findings to humans.

First, the progression of cirrhosis in animal models occurs over weeks, whereas human cirrhosis develops over years or decades, with multiple insults and fluctuating disease activity [82]. Second, animal models generally lack comorbidities prevalent in patients with cirrhosis, such as alcohol consumption, metabolic syndrome, or cardiac risk factors, which significantly influence myocardial function. Third, interspecies differences in cardiac electrophysiology, neurohumoral regulation, and myocardial architecture further complicate direct extrapolation. Moreover, most animal studies utilize young, genetically homogeneous subjects under controlled conditions—a stark contrast to the heterogeneous, often elderly, and medically complex human population.

A critical yet often overlooked obstacle in CCM research is the fundamental difference in cirrhosis etiology between humans and animal models.

Human cirrhosis is highly heterogeneous in cause and progression. Major etiologies include chronic viral hepatitis (HBV, HCV), alcohol-associated liver disease (ALD), non-alcoholic fatty liver disease/non-alcoholic steatohepatitis (NAFLD/NASH), autoimmune and cholestatic liver diseases, and genetic disorders (e.g., hemochromatosis, Wilson’s disease). Many of these factors can directly or indirectly affect the heart—for example, chronic alcohol misuse may cause alcoholic cardiomyopathy regardless of cirrhosis, while iron overload in hemochromatosis can induce restrictive cardiomyopathy. In contrast, experimental cirrhosis is typically induced by a single, controlled mechanism. Hepatotoxins such as carbon tetrachloride (CCl_4_) or thioacetamide (TAA) cause direct liver injury; bile duct ligation (BDL) models pure cholestatic fibrosis. Reliable ethanol-induced cirrhosis in rodents is difficult to achieve without co-toxins and rarely mimics the metabolic context of human ALD. Similarly, chronic viral hepatitis models are impractical in most species due to host barriers.

These etiological gaps matter. For example, CCM in NAFLD (non-alcoholic fatty liver disease)/NASH (non-alcoholic steatohepatitis) patients may overlap with metabolic cardiomyopathy driven by obesity, diabetes, and atherosclerosis—factors largely absent in standard rodent toxin models. BDL (bile duct ligature) models overrepresent cholestasis, which accounts for only a minority of cirrhosis cases in clinical practice. Such mismatches limit the direct applicability of experimental findings.

### Study Limitations

Among the limitations of our study, it is important to highlight the relatively limited number of studies conducted in this field over the past five years, as well as the fact that most of these investigations have been carried out on animal models.

There are also other limitations that should be acknowledged. First, the extrapolation of findings derived from animal models to human patients with cirrhosis is difficult and may affect the translational applicability of certain pathophysiological pathways. Second, the paucity of high-quality longitudinal data constrains our ability to fully elucidate the natural history and progression of CCM, as well as its potential prognostic implications over time. Finally, the absence of standardized and universally accepted diagnostic criteria for CCM contributes to substantial variability across studies, hindering meaningful comparisons between cohorts and complicating the development of consistent, evidence-based clinical management strategies. These constraints underscore the need for well-designed clinical studies involving human subjects, with a comprehensive assessment of all implicated pathophysiological mechanisms. A thorough understanding of these mechanisms could pave the way for the development of novel therapeutic strategies targeting this still insufficiently understood condition.

## 5. Conclusions

The mechanisms responsible for the development of CCM are complex and not yet fully understood. Both local immune responses and parenchymal alterations within the heart, triggered by the systemic stress associated with cirrhosis, may contribute to the development of cardiac dysfunction. Further animal studies and clinical trials are necessary to improve our understanding of the pathophysiology of this condition. A precise elucidation of these mechanisms may lead to the identification of targeted therapeutic strategies, ultimately improving the prognosis for affected patients.

## Figures and Tables

**Table 1:** Main original articles on CCM pathophysiological mechanisms published in the last 5 years.

Clinical Study and Authors	Year/Number of Subjects	Authors	Conclusions
Studies on Humans			
Cirrhosis Promotes Cardiac Fibrosis Development by Inhibiting Notch1 in Cardiac Fibroblasts	2025/*n* = 90	Sun et al. [64]	Cirrhosis-induced high levels of circulating TGF-β1 increase the progression of cardiac fibrosis through the inhibition of Notch1 in a DNA methylation-dependent mechanism. These findings provide insights into the development of preventative measures for cirrhosis-induced cardiac fibrosis risk
Role of Anti-Beta-1-Adrenergic Receptor Antibodies in Cardiac Dysfunction in Patients with Cirrhotic Cardiomyopathy	2021/*n* = 352	Ma et al. [29]	Anti-β1-AR levels in the CCM group were significantly higher than those in the non-CCM group Anti-β1-AR is a useful predictive biomarker for the presence of CCM and eventually may also have therapeutic implications
Studies on animals—mice/rats			
The role and mechanism of P2X7R in cirrhotic cardiomyopathy	2024/mice (*n* = N/A)	Shao et al. [65]	Targeting and inhibiting the expression of P2X7R not only alleviated myocardial inflammation and apoptosis but also enhanced cardiac performance
Protective role of the CD73 (5’-nucleotidase, ecto-5’-nucleotidase)/A2AR3 axis in cirrhotic cardiomyopathy through negative feedback regulation of the NF-κB pathway	2024/mice (*n* = N/A)	Zhao et al. [66]	CD73 (5’-nucleotidase, ecto-5’-nucleotidase)/A2AR signaling axis mitigates myocardial inflammation and apoptosis induced by cirrhosis
Galectin-3 inhibits cardiac contractility via a tumor necrosis factor alpha-dependent mechanism in cirrhotic rats	2022/rats (*n* = 24)	Yoon et al. [67]	Increased Galectin-3 in the cirrhotic heart significantly inhibited cardiac contractility
Silymarin Administration Attenuates Cirrhotic-induced Cardiac Abnormality in the Rats: A Possible Role of β_1_-adrenergic Receptors and L-type Voltage-Dependent Calcium Channels	2022/rats (*n* = 32)	Bayat et al. [68]	Significant up-regulation of ventricular β_1_-AR and L-VDCC Cardiac expression of the β_1_-AR and L-VDCC was down-regulated toward normal values by either higher or lower doses of the silymarin extract
Myocardial Dysfunction in Cirrhotic Cardiomyopathy is Associated with Alterations of Phospholamban Phosphorylation and IL-6 Levels	2021/rats (*n* = 30)	Gregolin et al. [69]	CCM is associated with decreased cardiac contractility with alteration of phospholamban phosphorylation in association with higher cardiac pro-inflammatory IL-6 levels
Autonomic regulation of imbalance-induced myocardial fibrosis and its mechanism in rats with cirrhosis	2021/rats (*n* = 40)	Yu et al. [70]	The levels of pro-inflammatory factor IL-6 were increased, whilst the expression of β1-AR protein and muscarinic M2 receptor was decreased in rats with cirrhosis, suggesting that abnormal inflammatory reaction, autonomic regulation, and other mechanisms may be involved in cirrhosis-related damage to the myocardium and accelerate ventricular remodeling
Impaired myosin isoform shift and calcium transients contribute to cellular pathogenesis of rat cirrhotic cardiomyopathy	2020/rats (*n* = N/A)	Honar et al. [71]	Impaired myosin isoform shift and calcium transients are important contributory mechanisms underlying the pathogenesis of the heart failure phenotype seen in cirrhosis.
Interplay of Liver-Heart Inflammatory Axis and Cannabinoid 2 Receptor Signaling in an Experimental Model of Hepatic Cardiomyopathy	2020/mice (*n* = N/A)	Matyas et al. [72]	Treatment of cirrhotic mice with a selective cannabinoid-2 receptor (CB_2_-R) agonist attenuated inflammation and fibrosis

anti-β1-AR—anti-β_1_-adrenergic receptor antibodies; L-VDCC—L-type voltage-dependent calcium channels; A2AR—A2 type adenosine receptor; IL-6—Interleukin 6; TGF-β1—transforming growth factor-β1; DNA—Deoxyribonucleic acid. N/A means not applicable. The number of animals is not specified in the articles.

## Data Availability

The dataset is available on request from the authors.

## Abbreviations

The following abbreviations are used in this manuscript:

A2AR	Adenosine A2 receptor
ATP	Adenosine triphosphate
BNP	Brain natriuretic peptide
cAMP	Cyclic adenosine monophosphate
CCM	Cirrhotic cardiomyopathy
CCC	Cirrhotic Cardiomyopathy Consortium
cGMP	Cyclic guanosine monophosphate
CO	Carbon monoxide
ELISA	Enzyme-linked immunosorbent assays
GAL-3	Galectin-3
GLS	Global Longitudinal Strain
HC	Hepatic cirrhosis
IL 1β	Interleukin 1 beta
IL-6	Interleukin 6
LVDD	Left ventricular diastolic dysfunction
MAPK	Mitogen-activated protein kinases
MSC	Mesenchymal stem cells
NO	Nitric oxide
NOS	Nitric oxide synthase
RAAS	Renin-angiotensin-aldosterone system
SNS	Sympathetic nervous system
TNFα	Tumor necrosis factor alpha
